# Acknowledgement to Reviewers of *Materials* in 2015

**DOI:** 10.3390/ma9010066

**Published:** 2016-01-21

**Authors:** Materials Editorial Office

**Affiliations:** MDPI AG, Klybeckstrasse 64, CH-4057 Basel, Switzerland; materials@mdpi.com

The editors of *Materials* would like to express their sincere gratitude to the following reviewers for assessing manuscripts in 2015.

We greatly appreciate the contribution of expert reviewers, which is crucial to the journal’s editorial decision-making process. Several steps have been taken in 2015 to thank and acknowledge reviewers. Good, timely reviews are rewarded with a discount off their next MDPI publication. By creating an account on the submission system, reviewers can access details of their past reviews, see the comments of other reviewers, and download a letter of acknowledgement for their records. In addition, MDPI has launched a collaboration with Publons, a website that seeks to publicly acknowledge reviewers on a per journal basis. This is all done, of course, within the constraints of reviewer confidentiality. Feedback from reviewers shows that most see their task as a voluntary and mostly unseen work in service to the scientific community. We are grateful to our reviewers for the contribution they make.

Abbes, B.
Abdelkefi, Abdessattar
Abdelmalak, Michael N.
Abdessalem, A. Ben
Abenojar, J.
Abou Neel, Ensanya A.
Abraham, Wolf-Rainer
Acremann, Yves Marc
Affolter, Christian
Afzal, Adeel
Aggelis, Dimitrios G.
Agrela, F.
Aguayo, Andrés Tomás
Aguey-Zinsou, K.
Ahmad, Shahzada
Ahmed, Mirza Foisal
Ahn, Kyung Hyun
Aicher, Simon
Aidala, Katherine
Akazawa, Housei
Akhil, Garg
Al-Haik, Marwan
Al-Nawas, Bilal
Al-Sabbagh, Mohanad
Al-Tabbaa, Abir
Alabastri, Alessandro
Alam, M. Shahria
Alcoutlabi, Mataz
Alford, T.L.
Alford, Patrick W.
Alhwaige, Almahdi A.
Ali, Majid
Allen, Thomas
Alleno, Eric
Almqvist, Bjarne
Alonso, M.M.
Aman, Sergej
Amaro, A.M.
Amendola, Vincenzo
Amineh, Reza Khalaj
Amiruzzaman, Md
Amo-Ochoa, Pilar
Del Barco Carrion, Ana, J.
Anac, Ilke
Anastasiou, Eleftherios
Andreucetti, Noemi A.
Angelici, Robert J.
Angelov, Borislav
Anjos, Ofélia
Annamdas, Venu G.M.
Ansell, Martin P.
Antoine, Rodolphe
António, J.
Ao, Zhimin
Aparicio, Conrado
Arao, Yoshihiko
Arbelaiz, Aitor
Ardanuy, Mónica
Ardebili, Haleh
Aref, Amjad J.
Arena, Francesco
Arias Sánchez, Pedro
Ariga, Katsuhiko
Arima, Kenta
Armijo, Kenneth M.
Arnold, Donna C.
Arora, Hari
Arrieta, Marina
Arsecularatne, J.A.
Asamoto, Shingo
Ashiuchi, Makoto
Asthana, Rajiv
Athanasopoulou, A.
Atkinson, Alan
Aubert, Thierry
Avakian, Artjom
Avolio, Albert
Aymerich, Francesco
Azad, Saeid Kazemzadeh
Babicheva, Viktoriia
Badea, Ildiko
Badogiannis, E.
Bae, S.H.
Baeg, Kang-Jun
Baek, Seung-Hak
Bagby, Michael
Bai, Qian
Baikie, Tom
Baino, Francesco
Bajdich, Michal
Bakeer, Tammam
Baker, R. Tom
Baldoni, Matteo
Balonis, Magdalena
Bankiewicz, Dorota
Bannwarth, Markus B.
Bardella, Lorenzo
Barhoum, Ahmed
Barkey, Mark
Barranco, Angel
Barrio, Roberto
Bartasyte, A.
Bartocci, Pietro
Barua, Sutapa
Basalo, F.J. De Caso Y
Bäßler, Ralph
Bastidas-Arteaga, E.
Bäumler, Hans
Bayat, Alireza
Bayer, Ilker S.
Beake, Ben
Bechelany, Mikhael
Bella, Federico
Beller, Daniel
Bellucci, Francesco
Beltrán, M.
Bencardino, Francesco
Benmokrane, Brahim
Benniston, A.C.
Berardi, Valentino Paolo
Bergamin, Marco
Berger, Walter
Berger, Paul R.
Bergum, Kristin
Beun, Sébastien
Bianco, Vincenzo
Bickel, Jessica
Bideau, Jean Le
Bignozzi, Maria Chiara
Bihamta, Reza
Bikiaris, Dimitrios
Bikowski, André
Birbilis, Nick
Birkett, Martin
Biro, Elliot
Bishay, Peter L.
Blackman, Christopher
Blanco, Ignazio
Boerckel, Joel
Bogas, J. Alexandre
Bohner, Marc
Bolíbar, María Fenollera
Bolotov, Leonid
Bonet, M.
Bongiovanni, Roberta
Bönisch, Matthias
Bonnefond, Audrey
De Boor, J.
Borchers, C.
Bordelon, Amanda
Borges, João
Boria, Simonetta
Bormashenko, Edward
Borodianskiy, Konstantin
Bos, Jan-Willem
Bose, Ranjita K.
Bosshardt, Dieter D.
Botsis, John
Boudart, B.
Bowden, Mark
Branco, Fernando
Brantley, William A.
Braxtan, Nicole Leo
Brenner, Thomas J.K.
Brighenti, Roberto
Brigo, Laura
Bru, Kathy
Bruck, Hugh A.
Brueckner, R.
Brüning, Karsten
Brunner, Andreas J.
Brunone, Bruno
Buergers, Ralf
Buividas, Ricardas
Burkel, Eberhard
Caballero, Alvaro
Cabanelas, Juan Carlos
Cabrales, Luis
Cacciotti, Ilaria
Cachim, Paulo B.
Cadenaro, Milena
Cai, Gaochuang
Calderón, Verónica
Calnan, Sonya
Calogero, Guiseppe
Calvo, Begoña
Calvo-Guirado, José Luis
Calzolari, Arrigo
Cambier, Francis
Camões, A.
de la Campa, José G.
Campbell, Charles T.
Campbell, John
Cancelliere, Piergiacomo
Candela, Pietra
Canela, Maria
Cano-Barrita, P.F. De J.
Cantele, Giovanni
Capelli, Claudio
Caporali, Stefano
Cardoso, Elsa
Carlone, Pierpaolo
Carman, Greg
Carmona-Martínez, A.A.
Carnie, Matthew
Caron, Etienne J.F.R.
Carro-Lopez, Diego
Carroccio, S.C.
Caruntu, Gabriel
Casalino, Giuseppe
Castano, Carlos E.
Castillo-Martinez, E.
Castñeiras, Alfonso
Castro-Gomes, Joao
Castrucci, Paola
Catauro, Michelina
Cava, Robert J.
Cavalline, Tara
Celano, Umberto
Cerqueira, M.Â.P.
Cerveny, Silvina
Cesano, Federico
Chadwick, R.
Chan, K.S.
Chang, Chih-Hung
Chang, Ting-Chang
Chang, Yu-Chao
Chao, Sao-Jeng
Charalampakis, A.E.
Charpentier, Thibault
Chase, George G.
Chen, M.
Chen, Liang
Chen, Zhigang
Chen, Xiao-Bo
Chen, Xi
Chen, Xiaoyin
Chen, Shuo
Chen, Fang-Chung
Chen, Zhizhong
Chen, Wei-Ting
Chen, Lung-Chien
Chen, Xiaojian
Chen, Jem-Kun
Chen, Changle
Chen, Tao-Hsing
Chen, Biao
Chen, Yan
Chen, Cheng-Sao
Chenal, Jean-Marc
Cheng, Xingguo
Cherman, Vladimir
Cheung, Chin Li
Cheung, Wing-Hoi
Chi, Hang
Chiachio, Manuel
Chiang, Anthony S.T.
Chiang, Chin-Lung
Chiang, Joseph F.
Chiang, Kin-Seng
Chiba, Takayuki
Chikan, Viktor
Chirumbolo, Salvatore
Chmiel, Jarosław Leszek
Cho, Yong Soo
Cho, Dong-Woo
Cho, H.
Cho, YongJin
Choi, Yoon-Seok
Choi, Hyun Chul
Choi, Joshua J.
Choi, Hyoung Jin
Choi, Jin-Ho
Choi, Hyo-Jick
Chou, Jung-Chuan
Chou, Joshua
Chowdhury, Dibakar
Chu, Shidong
Chu, Hailiang
Chuang, Steven S.
Chui, Ying Hei
Chun, Youngjae
Chung, Chen-Kuei
Chung, Yong-An
Chung, Hoyong
Ciampi, Simone
Cifelli, M.
Cinca, N.
Ciocia, Alessandro
Ciofani, Gianni
Ciríaco, Lurdes
Ciric-Marjanovic, G.
Cirillo, Giuseppe
Claire Lepage, Anne
Clarke, Edmund
Cobas, Enrique
Cobo, Alfonso
Coelho, João M.P.
Cole, Mathew
Coll, Mariona
Concheiro, Angel
Conde, João
Conti, Michele
Cook, Robert F.
Coop, Matthew Richard
Coppieters, Sam
Corbu, Ofelia
Corradi, Marco
Corriou, Jean-Pierre
Cortie, Michael B.
Covas, J.A.
Craciun, Monica
Craeye, Bart
Cragg, Peter J.
Creatore, M.
Criado, M.
Cristiani, Pierangela
Critchley, Kevin
Croccolo, Dario
Cui, Fangsen
Curran, Declan J.
Czujko, T.
Dai, Jian-Guo
Dankers, Patricia Y.W.
Daoud, Walid
Das, Narottam
David, Bill
Davidson, Bruce A.
Davis, Fred
De Aza, Piedad N.
De Brito, Jorge
de Dios Rivera, Juan
De Peppo, Giuseppe M.
De Riccardis, Federica
De Rooij, Mario
De Smet, Louis C.P.M.
De Windt, Laurent
De With, G.
Dean, David
Dean, Julian
DeAngelis, Dominick
Decoster, Mark
Deitzel, Joseph M.
Del Fabbro, Massimo
del Pino, Ángel Pérez
Demirci, Ibrahim
Demis, Sotiris
Dentino, Andrew R.
Deotare, Parag
Desvergne, Jean Pierre
Devine, Declan M.
Dewan, M. Washim
Dhakal, Tara
Dhar, P.
Di Maio, Luciano
Dias, Salvador
Díaz-Calleja, Ricardo
Dickert, Franz L.
Dietrich, Dagmar
Dimopoulos, T.
Dirrenberger, Justin
Disfani, M.M.
Disfani, Mahdi Miri
Dixon, David A.
Dobrzański, L.A.
Dodds, Chris
Dogan, Fatih
Doherty, Andrew
Domingo, Concepción
Dominguez, J.
Dong, Mingdong
Dong, Hong
Dopazo, Gonzalo Astray
Doquet, V.
Dos Santos, M. Serge
Dos Santos, Rui Galhano
Dossou, Kokou
Douglas, Timothy E.L.
Driver, Julian
Drozd, Vadym
Duarte, Isabel
Dubey, Ashish
Duczek, Sascha
Duerkop, Axel
Dunai, László
Duncan, Warwick
Duong, Hai M.
Albuquerque, M.T.D.
Durante, Christian
Durojaiye, Tolulope
Durual, Stéphane
Dyskin, Arcady
Dziubla, Thomas
Edalati, Kaveh
Eik, Marika
Einarsrud, Mari-Ann
Eisenlohr, Philip
Eklund, Per
Ekvall-Hansson, Eva
El Sawi, Ihab
El-Kaderi, Hani M.
El-Lateef, Hany M. Abd
El-Mowafy, Omar
El-Zahab, Bilal
Elambasseril, Joe
Eliades, George
Eliyan, Faysal
Ellahi, Rahmat
Elliman, Jennifer
Endelt, Benny
Endo, Tatsuro
Endrodi, Balazs
Engler, Olaf
Erdem, Emre
Erjia, Liu
Esling, Claude
Esparza, P.
Esquivel-Upshaw, J.F.
Estellé, Patrice
Etzerodt, Anders
Evangelista, Luís
Fabbri, Paola
Fabio Zuluaga, Hector
Fabrizio, Quadrini
Fall, Mamadou
Fangueiro, Raul
Fantozzi, Gilbert
Farahani, Ali
Faria, Paulina
Faruqi, Mohammed A.
Fasl, Hubert
Feenstra, R.M.
Felner, Israel
Fernandes, P.R.
Fernandes, Emanuel M.
Fernandez, Ines
Ferone, Claudio
Ferreira, José M.F.
Ferreira, Placid M.
Fiedler, Thomas
Filinchuk, M. Yaroslav
Filippini, Mauro
Filippova, Maria
Fink, Gerhard
Fiore, V.
Fischer, Franz Dieter
Fischer, Holger
Flandin, L.
Fletcher, Ashleigh
Flint, Madeleine
Florczyk, Stephen J.
Florek, Justyna-Agata
Focacci, Francesco
Follain, Nadège
Fonollosa, Jordi
Foot, Peter
Fornasiero, Paolo
Foster, E. Johan
Foti, Dora
Frame, Molly
Frankel, Gerald S.
Fratini, Livan
Freed, Alan D.
Friák, Martin
Frick, Kevin K.
Fricke, Wolfgang
Friedrich, Joachim
Frigione, Mariaenrica
Frömling, Till
Fromm, Katharina
Frusteri, Francesco
Fu, Yin-Chih
Fukuda, K.
Furukawa, Rei
Furushima, R.
Gadre, Anand
Gahlert, Michael
Galanakis, Charis
Galassi, Carmen
Galca, A.C.
Galfetti, Luciano
Gallagher, John
Gallimard, Laurent
Gamstedt, Kristofer
Gandolfi, Maria G.
Ganser, Christian
Gao, Yuan
Garbuzenko, Olga
Garcés, Pedro
Garcia, Hermenegildo
Garcia, Aitzol
García Navarro, Justo
Garcia-Escorial, A.
Garcia-Godoy, Franklin
García-Márquez, Alfonso
Garcia-Moreno, Francisco
Garino, Claudio
Garralda, María A.
Gary-Bobo, Magali
Geddis, Demetris L.
Gei, Massimiliano
Gentile, Piergiorgio
Gerbig, Yvonne B.
Gerhardt, Holger
Ghassemali, Ehsan
Ghiotti, A.
Ghosh, Anindya
Ghrib, Faouzi
Giersig, Michael
Gigante, Giovanni E.
Gil, Francisco Javier
Gil, A.
Gil, Lluís
Gilbert, M.R.
Gille, P.
Giraud, Stephane
Girault, Hubert
Girousi, Stella
Gizdavic-Nikolaidis, M.R.
Gloria, Antonio
Głowacki, Eric Daniel
Godar, D.E.
Goddard, Julie M.
Godinho, Maria
Goel, Saurav
Goh, Suat Hong
Goldstein, Aaron S.
Gomart, Hector
Gong, Pan
Goñi, Asier
de las Nieves González, M.
González-Benito, Javier
González-Fonteboa, Belén
Goodall, Russell
Goodier, Chris
Goodwin, Andrew P.
Gorash, Yevgen
Gorfman, Semën
Gotoda, Hironobu
Gowayed, Y.
Gower, Laurie B.
Gracewski, S.M.
Graeve, Olivia
Granet, Robert
Granja, Juan R.
Grant, E.R.
Grasley, Zachary
Gravenkamp, Hauke
Graziani, Andrea
Greco, Antonio
Gregorczyk, Keith
Gregory, Richard L.
Grimm, Alejandro
Grinberg, Marek
Grinter, David
Grishchuk, Sergiy
Groche, Peter
Gruber, Reinhard
Gualandi, Chiara
Guan, Dongsheng
Gude, Veera Gnaneswar
Gudmarsson, Anders
Guedes, Mafalda
Guénin, Erwann
Guentsch, Arndt
Guerra, Gaetano
Guerrero, Miguel
Guesmi, Hazar
Guilmeau, Emmanuel
Gulino, Antonino
Guney, Durdu
Guo, Youguang
Guo, Fenghai
Guo, Zhanhu
Guo, Peixuan
Gupta, S.
Gupton, B. Frank
Gurdal, A. Erkan
Gurman, Pablo
Guth, Ulrich
Gutierrez, Junkla
Gutiérrez-González, C.F.
Gwenin, Christopher
Haaparanta, Anne-Marie
Habert, Guillaume
Hadad, Yakir
Haddadi, Farid
Hadjikakou, Sotiris K.
Haghdoost, Atieh
Hahn, Byung-Dong
Hahnel, Sebastian
Haider, Waseem
Hale, W. Micah
Ham, Hyung Chul
Hamada, Hidenori
Hamdy, Reggie C.
Hamilton, Carter
Han, Daewoo
Han, Guo-Cheng
Han, Yosep
Han, Jung-Suk
Han, Jeon Geon
Han, Tao
Han, Min-Cheol
Han, Ke
Han, Yousoo
Hanaor, Dorian
Handge, Ulrich A.
Hane, Kazuhiro
Hangai, Yoshihiko
Hänninen, Hannu
Hara, Masayuki
Hariri-Ardebili, M.A.
Haro-Dominguez, Pablo
Harris, Philip
Harrison, William
Harrison, Walter A.
Hashimoto, Masanori
Haslauer, Carla M.
Hassan, Marwa
Hassanein, Ahmed
Hassani-Gangaraj, S.M.
Hatamleh, Muhanad
Hatano, Tsutomu
Haverkamp, Richard G.
Haward, Simon
Hawk, J.A.
Hayakawa, Tohru
Hayashi, K.
He, Jingwei
He, Ping
Hector, Andrew L.
Hedayati, Mehdi K.
Heim, K.
Heindl, Ranko
Hellmich, Christian
Helth, Arne
Henning, Theunis F.P.
Heon, Heon
Heras, Angeles
Hernandez, Alvaro G.
Hess, Dennis W.
Hey, Jeremias
Highland, Matthew J.
Hill, Michael
Hill, Craig L.
Hilloulin, Benoit
Hilton, Harry H.
Hintzen, H.T.J.M.
Hiramatsu, Hidenori
Ho, Wen-Jeng
Ho, Johnny
Hoa, Suong V.
Hochrainer, Thomas
Hofmann, Douglas C.
Hofstetter, Wilhelm
Hohn, Keith
Holappa, Lauri
Holcomb, M.
Holdsworth, Stuart
Holmes, Niall
Holschemacher, Klaus
Holt, Rune M.
Holtz, Ronald L.
Hong, Dae Ho
Hong, Chang-Hee
Hong, Youngkeun
Hong, Liang
Hong, Woong-Ki
Honkanen, Seppo
Hoogenboom, Richard
Horiuchi, Yu
Hort, Norbert
Houng, Boen
Housecroft, Catherine E.
Hover, Kenneth Clark
Howard, Gary
Howell, Bob
Hristoforou, Evangelos
Hsiao, Kuang-Ting
Hsu, Jui-Ting
Hsu, Quang-Cherng
Hsu, Fu-Yin
Hsu, Cheng-Hsun
Hsu, Ching-Chi
Hsu, Jin-Chen
Hu, Anming
Hu, Jinlian
Huang, Haw-Ming
Huang, Po-Hao Adam
Huang, Haitao
Huang, Wu-Jang
Huang, Huang-Chiao
Huang, Jianwei
Huang, Chi-Yen
Hubert, O.
Hudhomme, Piétrick
Hug, Eric
Hughes, Larry
Huh, Jung-Bo
Huh, Yung
Hühne, Ruben
Hung, Chang-Mao
Hutchings, Lian
Hutchison, James
Hwang, Chao-Lung
Hwangbo, Chang Kwon
Ie, Yutaka
Igari, T.
Ikeda, Masahiko
Imperia, Paolo
Iniesta, Jesús
Inzana, Jason A
Ionescu, Andrei
Ionescu, Mihail
Irie, Masao
Isaad, Jalal
Ishihara, Atsushi
Ita, Kevin
Ivanov, Dimitri A.
Ivanov, Ilia N.
Ivey, Douglas
Iwamoto, Takeshi
Iwata, Rintaro
Iwata, Takanori
Jack, David
Jager, W.F.
Jajam, Kailash C.
Jakubowicz, Ignacy
Jalali, Said
James, Aaron
Jamshidinia, M.
Jan, Jeng-Shiung
Janda, Daniel
Jang, Soohwan
Jang, Seung-Yup
Jankowska-Sumara, Irena
Janolin, Pierre-Eymeric
Janus, Magdalena
Jaroniec, Mietek
Javed, Muhammad A.
Jayaseelan, Daniel Doni
Jayasinghe, Suwan
Jeng, Tzer-Ming
Jeon, Seokwoo
Jeong, S.-J.
Jeong, Mi-Yun
Jepsen, Julian
Jha, Kshitij C.
Ji, Gang
Ji, Wei
Ji, Vincent
Jia, Rui
Jiang, Qinghui
Jiang, Xian
Jiao, Yan
Jimenez, Catalina
Jimenez, Ana Eva
Jiménez, Alberto
Jiménez-Cruz, Federico
Jimenez-Rojo, Lucia
Jin, Kejia
Jin, Fei
Jo, Young Min
Jobic, Stéphane
Joly, Nicolas
Jones, Steve
Jones, Jacob L.
Jood, Priyanka
Joos, Jonas
Joshi, Giri
Jourani, A.
Jørgensen, Mikkel
Juenger, Maria C. Garci
Jun, Youn Chul
Jun, Ho-Wook
Jun, Martin
Jung, Gun Young
Kafizas, Andreas
Kahouli, Abdelkader
Kahr, Bart
Kakkar, Ashok K.
Kalantar Zadeh, Kourosh
Kamal Mohamed, S.M.
Kan, Chi-Wai
Kanaan, Hani
Kanazawa, Manabu
Kaneko, Yoshihisa
Kanellopoulos, Antonios
Kaneti, Yusuf
Kang, Lifeng
Kang, Junsuk
Kang, Sang Ook
Kang, Hyun Wook
Kang, Y.C.
Kang, Jeung Ku
Kannan, Sukeshwar
Kantamaneni, Komali
Karadelis, John N.
Karalekas, Dimitris
Karp, Jeffrey M.
Karpinsky, D.V.
Karpukhina, Natalia
Kashaev, Nikolai
Kasper, Gerhard
Katz, Jonathan I.
Kaufman, Laura J.
Kaur, Gurbinder
Kawanago, Takamasa
Kawase, Tomoyuki
Kawashima, Shiho
Kazemian, Hossein
Kearney, Cathal
Keller, Valérie
Kennedy, Andrew
Keogh, Michael
Kern, Matthias
Kerscher, Eberhard
Kevadiys, Bhavesh
Khabibullin, Amir
Khaliq, Wasim
Khan, Abdul A.
Khandaker, Morshed
Khang, Dahl-Young
Khaykovich, Boris
Khun, Nay Win
Kidoaki, Satoru
Kiener, Daniel
Kietzig, Anne-Marie
Kikuchi, Y.
Kim, Jin Kuk
Kim, Jang-Ho Jay
Kim, JunHee
Kim, Woochul
Kim, Young
Kim, Seong
Kim, Kwang-Mahn
Kim, Dong-Ho
Kim, Jun-Hyun
Kim, Kang-Su
Kim, Myung-Joo
Kim, Ji-Hwan
Kim, Donghyun
Kim, Jaheon
Kim, Sung Jae
Kim, Youngkyoo
Kim, Hak-Yong
Kim, Sang Wan
Kim, Yoong Ahm
Kim, Seonah
Kim, Dongjoo
Kim, Ji-Hee
Kim, Seong Yun
Kim, Dae Eun
Kim, Byung-Sun
Kim, Seong Keun
Kim, Hak Yong
Kim, Jihoon
Kimura, Yoshiharu
King, Peter
Kinuthia, John
Kirihara, Soshu
Kitajou, Ayuko
Kitazono, Koichi
Kiyobayashi, Tetsu
Klar, Thomas A.
Klinge, U.
Knaster, J.
Knezevic, Marko
Knowles, Kathryn
Ko, Seung Hwan
Kobayashi, Satoshi
Koduru, Janardhan R.
Koga, Hirotaka
Koh, W.S.
Koh, Young-Hag
Köhler, Jürgen
Koida, Takashi
Kolesnichenko, V.L.
Komasa, Satoshi
Komnitsas, K.
Komori, Kikuo
Kondori, Babak
Kong, Ling Bing
Konsolakis, Michalis
Koppel, Kadri
Kosaka, Tatsuro
Koskinen, Pekka
Kostopoulos, V.
Kotadia, Hiren
Kotobuki, Masashi
Kotsakis, Georgios A.
Kourkoutas, Yiannis
Kraus, Tobias
Krause, Andreas
Kroll, Stephen
Krstulovic Opara, Lovre
Krumeich, Frank
Krupski, Aleksander
Kubiak, Krzysztof J.
Kumada, Nobuhiro
Kumar, Golden
Kumar, Shailesh
Kumar, Vijay
Kumar, Sudhir
Kumbar, Sangamesh G.
Kundu, Santanu
Kuo, Dong-Hau
Kuo, Chao-Yin
Kuo, Chil-Chyuan
Kuo, Shiao-Wei
Kuo, Wen-Ten
Kupka, Marian
Kuramoto, Koji
Kurosaki, Ken
Kurt, Mehmet
Kurumisawa, Kiyofumi
Kusano, Yukihiro
Kwan, James
Kwek, J.W.
Kwok, Harry
Kwon, Tae-Yub
Kyzioł, Agnieszka
La Cascia, Marco
La Cognata, M.
La Mantia, Francesco P.
La Mantia, F.P.
Labidi, Jalel
Lad, Robert
Lahiri, Abhishek
Lam, Frank
Lam, Kwok-Ho
Lanceros-Mendez, S.
Lannitti, D.A.
Laquerriere, Patrice
Laroche, Céline Laroche
Laukkanen, Pekka
Laurent, Jean Paul
Lauria, A.
Lavertu, Marc
Lavina, Sandra
Lawrence, Joseph G.
Lazaro Garcia, Alberto
Le, Ha Thanh
Le Breton, Jean-Marie
Le Tien, Canh
Leardini, Fabrice
Lebedev, Oleg I.
Leblanc, Roger
Lecomte, Philippe
Lee, Dongwon
Lee, W.
Lee, Sanboh
Lee, Bor-Shiunn
Lee, Y.
Lee, Bongmook
Lee, Jong-Sub
Lee, Koon-Yang
Lee, Jungwoo
Lee, Jusang
Lee, J.S.
Lee, Jae-Shin
Lee, Kwang-Myong
Lee, Hsien Hua
Lee, Jun-Jae
Leemann, Andreas
Léger, Bastien
Léger, Pierre
Lehmhus, Dirk
Leiza, Jose R.
Lemarchand, Claire
Yu, T. Leon
Lépinay, Sandrine
Lepoittevin, Bénédicte
Letizia, Rosa
Levänen, Erkki
Levitas, Valery I.
Lewandowski, John
Lewis, Gladius
Lewis, David
Li, Li
Li, Robert K.Y.
Li, Longyuan
Li, Guoqiang
Li, Yi
Li, Luping
Li, Zheng
Li, Song
Li, H.
Li, Xiaojun
Li, X.Y.
Li, Yanjun
Li, Jiantong
Li, H.-T.
Li, Xiangyu
Liao, Ting
Liao, Shu-Chuan
Liao, Susan
Libera, Joseph A.
Licheri, Roberta
Liew, S.L.
Liewald, Mathias
Lightfoot, Philip
Lignola, Gian Piero
Liguori, B.
Limodin, Nathalie
Lin, Chun-Ming
Lin, Weiwei
Lin, Ching Hsuan
Lin, I.-Nan
Lin, Yuan-Ching
Lin, Wei-Ting
Lin, Leyu
Lin, Kuei C.
Lin, Hung-Mao
Lin, Far-Ching
Lin, Chia-Feng
Lin, Yuyuan
Lin, Hong-Ping
Lin, Jiann-T'suen
Linder, Christian
Lindström, Tom
Ling, Jian
Ling, Bo
Ling, Maurice Ht
Linko, Veikko
Liu, Yuqing
Liu, Chuan-Pu
LIU, Hongwei
Liu , Xiaohang
Liu, Fuqiang
Liu, Jinyun
Liu, Yanjun
Liu, Shih-Jung
Liu, Rui
Liu, Ming
Liu, Qimao
Liu, Yifan
Lloyd, Natalie
Lo, Fang-Yuh
Lo, Chien-Fong
Lo Conte, A.
Locatelli, Clinio
Lodewijks, Kristof
Lohbauera, Ulrich
Lohmüller, Theobald
lombardi, M.
Lopes, Joao Marcelo J.
Lopes, S.M.R.
Lopes, Maria Ascensão
Lopéz-Ramon, Maria V.
Lorenz, Bernd
Losada-Perez, Patricia
Losch, Pit
Love, Gordon
Lozano, Karen
Lu, Li
Lu, Yao
Luan, Binquan
Luche, Jocelyn
Luches, Paola
Luengo, Gustavo
Lufaso, Michael
Luhrs, Claudia
Luong, Dzung Dinh
Lupascu, Tudor
Ma, Jeff
Ma, Deyun
Ma, Anxin
Ma, Yuan-Ron
Maas, M.
Maclachlan, Mark J.
Madian, M.
Maeyama, Katsuya
Maggioni, Daniela
Magliulo, Gennaro
Magniez, Kevin
Mahmoud, Firas
Maioli, Emanuela
Majumdar, Apala
Makabe, Chobin
Makihira, Seicho
Maleki-Dizaji, Saeedeh
Malfatti, Luca
Malladi, S.R.K.
Malucelli, Giulio
Maly, Kenneth E.
Mandracci, Pietro
Mangialardi, Teresa
Manso, Juan M.
Mansoori, G. Ali
Mante, Francis K.
Manzi, Stefania
Mao, Chuan-Bin
Marani, Debora
Marcal, Helder
Marinero, Ernesto E.
Markovic, Ljubisa
Markovich, Gil
Marrocchi, Assunta
Marszalek, Marta
Martel, S.
Marthinsen, Knut
Martin, T.J.
Martinelli, Enzo
Martinez, Enrique
Martínez Ramirez, S.
Martino, Sabata
Martins, Rodrigo
Maruyama, Takahiro
Marzani, Alessandro
Mascia, Leno
Masis, Monica Morales
Mason, Alex
Matias, Ignacio Raul
Matolín, Vladimír
Matsunaga, Naoki
Matsuno, Hisao
Mattia, Davide
Maurer, Thomas
Mavrokefalos, A.
May, Steven J.
Mayer, H.
Mayeur, Jason R.
Mazaj, Matjaž
Mazyck, David W.
McCarthy, Edward
Meda, A.
Mehl, Christian
Melati, Rabia
Melchels, Ferry
Melchers, Robert
Mele, Paolo
Melero, H.
Menaa, Bouzid
Mendis, Chamini
Meneghetti, Giovanni
Menezes, Pradeep L.
Mergia, Konstantina
Merino, Beatriz Sanz
Merlen, A.
Mertens, Christian
Mestre, Ana S.
Mestres, Lourdes
Metaxa, Zoi S.
Metelli, Giovanni
Miao, Jianwei
Michels, Julien
Mikael, Skrifvars
Milanese, Chiara
Milenkovic, Srdjan
Miller, Cheryl
Mills, David
Min, Kyung-San
Minamikawa, Hajime
Mingler, Bernhard
Minguzzi, Alessandro
Minteer, Shelley D.
Miotello, Antonio
Mirzahosseini, M.
Mishra, Yogendra K.
Misra, Devesh
Mitali, Kakran
Mitra, Ashim
Miyamoto, Manabu
Miyazaki, Toshiki
Modreanu, Mircea
Moghadam, Afsaneh D.
Mohammadi, Maysam
Mohanta, Antaryami
Mohn, Dirk
Monchau, Francine
Monje, Alberto
Monteil-Rivera, Fanny
Monteiro, Eliseu
Monti, Alessio
Montúfar, Edgar B.
Monzon, Eric V.
Moon, Young-Hoon
Moore, Jonathan E.
Moore, Gary
Moorefield, Charles N.
Moradian-Oldak, Janet
Morandeau, Antoine
Morasch, A.
Morent, Rino
Morgado, Jorge
Morgan, Alexander
Moriconi, Giacomo
Moriga, Toshihiro
Morimura, Shigeru
Morkoc, Hadis
Morreale, Marco
Morris, David L.
Morris, Michael
Mortensen, Andreas
Moseke, Claus
Moshaverinia, Alireza
Moslehi, Behzad
Mostafa, Ahmad
Moure, Alberto
Mugnaini, Veronica
Mukherjee, Abhijit
Müller, Bert
Müller, Gerhard
Müller, Urs
Muntean, Adrian
Münzenrieder, Niko S.
Muroyama, Masanori
Murphy, Ciara M.
Muszynski, Larry
Mysliwiec, Tami
Nabae, Yuta
Nair, Jijeesh R.
Naito, Yoshihito
Naji, Amar
Najm, Husam
Nakagaito, Antonio N.
Nakashima, T.
Nam, Ki-Woo
Narciso, Javier
Naser, Jamal
Nassar, Mohannad
Nassar, A.R.
Natali, Maria Elia
Natali, Franck
Natter, Harald
Navacerrada, M.A.
Navarro, M.M. Sánchez
Navarro, Carlos
Nedelec, Jean-Marie
Neenu Singh, Doak
Nei, Jean
Neises, Martina
Newman, Ward
Nganga, Sara
Ngo, Truc T.
Nguyen, Juliane
Ni, Meng
Ni, De Wei
Nichols, Joan E.
Nien, H.H.
Niesner, Daniel
Nikita, Bityurin
Nikolelis, Dimitrios P.
Nilges, Tom
Nishiwaki, Tomoya
Nisticò, Roberto
Nitin, Shukla
Nobile, Lucio
Nocetti, Michela
Nogales, Emilio
Nomani, M.W.K.
Nomura, Takahiro
Nonomura, Kazuteru
Noorani, Rafiqul I.
Normand, Bernard
Norquist, Alexander
Nouvel, Cécile
Novák, Pavel
Nowak, Bożena
Numata, Keiji
Nunes, Sandra
Nyström, Gustav
O'Connor, Ciaran P.J.
Obraztsov, Alexander
Ochiai, Tsuyoshi
Oesterschulze, E.
Ogilby, Peter R.
Ogris, Manfred
Oh, Se Heang
Oh, C.
Ohashi, Naoki
Okamoto, Masami
Okimoto, Yoichi
Olagnon, Christian
Oliveira, F.
Olszta, Matthew
Ono, Kanji
Onoue, Kozo
Orava, Jiri
Orbulov, Imre
Orecchio, Santino
Ourir, Abdelwaheb
Ouyang, Hao
Özcan, Mutlu
Ozmen, Mustafa
Pacheco-Torgal, F.
Pyttel, B.
Padovano, Elisa
Paipetis, A.S.
Palgrave, Robert
Pallav, Kumar
Pallozzi, Emanuele
Palmquist, Anders
Panchakarla, Leela
Pang, Xueyu
Papaccio, GianPaolo
Papoulis, Dimitrios
Parenti, Paolo
Parisi, Fulvio
Park, Young-Seok
Park, Yoonjee
Park, Young Jae
Park, Yeong-Joon Park
Park, Hyung-Ho
Park, Chan Eon
Park, Hwan-Man
Park, Sung Ha
Parlett, Christopher
Partridge, James G.
Pasetto, Marco
Pasetto, Pamela
Pasta, Mauro
Patra, Niranjan
Patra, Anirban
Patro, L.
Patten, John
Patterson, Brian M.
Pavlidis, Ioannis
Peixinho, Nuno
Pellegrino, Carlo
Pellicer, Eva
Pellinen, Terhi
Peluso, Paola
Peng, Cheng
Peng, Erwin
Pereira, Jefferson
Pereira, Letricia Barbosa
De Oliveira, L.A.P.
Pérez, Gloria
Pérez-Prado, M.T.
Perras, Matthew A.
Perrella, M.
Petter-Puchner, A.H.
Peyratout, Claire
Pfeifer, Carmem
Pham, Hien
Phan, Thang T.
Pichler, Bernhard
Pillai, Rajalekshmi C.
Pina , María Pilar
Pires, E.
Pisello, Anna
Piskin, Sabriye
Pistone, Alessandro
Pitsikalis, Marinos
Plamper, Felix
Plummer, John C.
Po-We, Kao
Pober, Richard
Podraza, N.J.
Poeppelmeier, K.R.
Pogatscher, S.
Poggio, Claudio
Polacco, Giovanni
Polvi, Jussi
Polydorou, Olga
Pons, Josefina
Pontikes, Yiannis
Poologanathan, K.
Poon, S.J.
Popat, Ketul C.
Populoh, Sascha
Potgieter, Herman
Poulet, L.
Poulikakos, Lily
Poyet, Stéphane
Pozza, Luca
Pradhan, Sangram K.
Pramanik, A.
Pratsinis, Harris
Pretsch, Thorsten
Prida, V.M.
Prieto, Julio
Proust, Gwenaelle
Provis, John L.
Prud’homme, R.E.
Psarras, Georgios
Pu, Yong-Jin
Pucci, Andrea
Pucha, Raghuram V.
Pucinotti, Raffaele
Pudasaini, Pushpa Raj
Puglia, Debora
Puiggalí, Jordi
Puligilla, Sravanthi
Pusch, Andreas
Pylypenko, Svitlana
Qasim, Saad
Qin, Zhao
Quadbeck, Peter
Quadrini, Fabrizio
Quirino, Rafael
Radecka, Iza K.
Raftery, Gary M.
Ragab, K.A.
Ragauskas, Arthur J.
Rahbar, Nima
Rahier, Hubert
Rakesh, Leela
Ramadass, Kavitha
Ramanavicius, Arunas
Ramos, Idalia
Ramulu, Mamidala
Rana, D.
Rana, Sohel
Rao, K. Venkat
Raub, Chris
Ravelli, Davide
Ray, Sekhar
Refait, P.
Regmi, Bishnu
Rehman, Ihtesham
Reichert, Christoph
Reilly, Gwendolen
Reimanis, Ivar
Reis, P.N.B.
Renò, Filippo
Renterghem, Wouter Van
Restivo, J.
Reverberi, Andrea
Reynolds, Mark A.
Riches, Philip E.
Rider, A.N.
Ridi, Francesca
Rimondini, Lia
Ritala, Mikko
Rittmann, Simon
Riu-Aumatell, M.
Riziotis, Christos
Robben, Lars
Robinson, Sara C.
Roby, Johanne
Rocha, Luciana
Rodella, Luigi Fabrizio
Rodríguez, Fernando
Roggendorf, Matthias J.
Rogl, Peter
Romana, Laurence
Romanoff, Jani
Rondinelli, James M.
Roper, D. Keith
Rosa, Vinicius
Rose, Marcus
Rosner, Mordechai
Rothenberg, Gadi
Rother, Astrid
Rouabhia, Mahmoud
Roudsari, Reza Vahid
Ruan, Gang
Rubanov, Sergey
Rubio-Marcos, F.
Rudzitis-Auth, J.
Ruggieri, Fabrizio
Ruhl, Aki Sebastian
Runge, Jude Mary
Rüscher, Claus
Rushing, Todd S.
Rusnati, Marco
Russo, Valeria
Ryu, Sung Hun
Saadatfar, Mohammad
Saafi, Mohamed
Sabbagh, Ahmed
Saccani, Andrea
Sacher, Edward
Saffar, K.P.A.
Safiuddin, M.
Saggu, Javed
Sah, Hongkee
Sakaguchi, Toshikazu
Sakaushi, Ken
Sakida, Shinichi
Salerno, Marco
Salmi, Mika
Salmon, Lionel
Salvia, Michelle
Samavedi, Satyavrata
Samulionis, Vytautas
Samy, Merabia
Samyn, Pieter
Sánchez-Silva, Luz
Sandak, Anna
Sandhaas, Carmen
Sangiorgi, Cesare
Sanjayan, Jay
Sanjuán, Miguel Ángel
Santos, Diogo M.F.
Santos, Lídia
Santos, Abel
Santos, Tírcia C.
Santos, Fábio André
Santulli, C.
Sapelkin, Andrei
Sara, Rios Silva
Sarasini, Fabrizio
Sarker, Prabir K.
Sato, Soshi
Sato, Yoichi
Sato, Hirotaka
Sauvage, Xavier
Sauvé, Geneviève
Savija, Branko
Savina, Irina
Saw, Kim Guan
Scarselli, Manuela
Schartel, Bernhard
Schauer, Caroline
Schenning, A.P.H.J.
Scherrer, Susanne S.
Schierholz, Roland
Schiffman, Jessica D.
Schirp, Arne
Schlaad, Helmut
Schmidt, Jochen
Schneider, Raphaël
Schwank, Johannes
Schwitalla, A.
Scribante, Andrea
Seantier, Bastien
Seebauer, G.
Seetharaman, S.
Segawa, Hiroyo
Seidman, David N.
Semaltianos, N.G.
Semboshi, Satoshi
Sen, Tapas
Seo, D.-G.
Seo, Dae-Shik
Seok, Hyun-Kwang
Serrano, Susana
Shah, Ramille
Shahrbaf, Shirin
Shahzad, Asim
Shao, Jian-Fu
Shao, Lin
Shapter, Joe G.
Sharif-Khodaei, Z.
Sharma, Suraj
Sharma, Yogesh
Shehata, Medhat
Sheikh, Zeeshan
Shen, Kelvin K.
Shen, Jianhu
Sherratt, Michael J.
Shi, Xun
Shi, Quan
Shi, Fanian
Shi, Jen-Bin
Shieh, Jiann
Shih, Wen-Pin
Shim, Changsu
Shimamura, Y.
Shin, Kwang-Bok
Shin, Ki-Hyuk
Shiraishi, Yasuhiro
Shiravand, Fatimeh
Shmulsky, Rubin
Shoji, Kakio
Shokouhi, Parisa
Shrestha, Lok Kumar
Shu, Xiang
Shupe, Todd F.
Sideridou, Irini
Sideris, Kosmas
Sier, Cornelis F.M.
Sierra, Teresa
Sierra, Miguel Angel
Sigusch, Bernd W.
Sikora, Karol
Silva, Tiago H.
Silva, João
Silverman, Ronald H.
Simmonds, Paul J.
Simpson, Garth J.
Sinclair, K.D.
Singh, Ranjan
Singh, Alok
Singh, G.
Skov, Anne
Slinker, Jason D.
Smått, Jan-Henrik
Smith, Lloyd M.
Smith, Nicholas I.
Smith, Rosemary L
Snead, Malcolm
Sobey, A.J.
Sogabe, T.
Somayazulu, M.
Son, Ji-Won
Son, David Y.
Song, G.
Song, Guang-Ling
Song, Leon
Song, Young Min
Song, Xu
Sonsteby, H.H.
Soos, Miroslav
Sora, Isabella Natali
Sorgente, Donato
Soroushian, Parviz
Sothmann, Björn
Spadea, Giuseppe
Speliotis, T.
Speranza, Giorgio
Spies, Benedikt C.
Spinella, Nino
Spychaj, Tadeusz
Srivatsan, T.S.
Srubar, W.V.
Stafford, Luc
Stamatakis, Michail
Stanton, Kenneth T.
Stathis, Bousias
Stathopoulos, A.T.
Stavrinadis, A.
Stavropoulos, P.
Stavroulakis, G.E.
Stawarczyk, Bogna
Steinbach, Ingo
Steinberger-Wilckens, R.
Steinhäuser-Andresen, S.
Stephens, John
Stevenson, Timothy
Stojek, Zbigniew
Stoliarov, S.I.
Stoll, Sarah L.
Strano, Michael S.
Stratakis, Emmanuel
Straumal, Boris
Strauß, Sarah
Strzhemechny, Yuri
Su, Chunming
Su, Chun-Yi
Suchorab, Zbigniew
Suchorski, Yuri
Sudo, Atsushi
Suemune, Ikuo
Sugawa, Kosuke
Sugawara-Narutaki, A.
Suh, Kwang S.
Suib, Steven L.
Sukhishvili, Svetlana
Sun, Chang-Jung
Sun, Y.F.
Sun, Kien Wen
Sun, Wenping
Sundaram, K.B.
Sundqvist, Jonas
Suplicz, András
Suryanto, Benny
Swain, Michael
Swihart, Mark T.
Swolfs, Yentl
Syrrokostas, G.
Tabaković, Amir
Tabata, Yasuhiko
Tabrizian, Roozbeh
Taha, I.
Tahraoui, Abbes
Tai, Masayuki
Takagi, Noriaki
Takagi, Youhei
Takahashi, Keisuke
Takashiri, Masayuki
Takoudis, C.G.
Taleb, Abdelhafed
Tam, Chat-Tim
Tamanoi, Fuyuhiko
Tammaro, Marco
Tan, Amy G.Y.
Tan, Kwek-Tze
Tancret, Franck
Tang, C.J.
Tang, P.
Tang, James Z.
Taniguchi, Masateru
Taniike, Toshiaki
Tanksale, Akshat
Tanner, Julian A.
Tannert, Thomas
Tanoue, Shuichi
Tapfer, Leander
Tas, A. Cuneyt
Tateiwa, Naoyuki
Tateo, Flaviano
Tauber, Andreas
Tauböck, Tobias
Tavangarian, F.
Tavares, Joao M.
Tay, Chor Yong
Teghil, R.
Teixeira, Fernando
Teixeira-Dias, F.
Temiz, Yuksel
Temnov, Vasily
Teng, Hsisheng
Teranishi, Takashi
Texter, John
Teyssier, Jeremie
Themistou, Efrosyni
Theocharopoulos, A.
Theohari, S.
Tian, Yuan
Tidehag, Per
Timalsina, Yukta P.
Timothy, Wangler
Ting, Simon
Tjin, Swee Chuan
Tognetti, Vincent
Tölli, Hanna
Toma, Ionut-Ovidiu
Tomashchuk, Iryna
Tomasi, Roberto
Tomich, John
Tomlinson, Douglas
Toppari, Jussi
Torres-Costa, V.
Torres-Lagares, D.
Torricelli, Fabrizio
Tosheva, Lubomira
Toubal, Lotfi
Toudert, Johann
Toupance, Thierry
Tozzini, Valentina
Trad, Tarek
Treccani, Laura
Trindade, Bruno
Tripathi, Jitendra K.
Tripodo, Giuseppe
Truss, Rowan
Tsai, Hsiao-Chung
Tsao, L.C.
Tsay, L.W.
Tsay, Chien-Yie
Tseng, I.-Hsiang
Tsoi, James Kit Hon
Tsoukalas, Dimitris
Tsui, C.P.
Tsunashima, Ryo
Tsuruoka, Takaaki
Tu, C.-S.
Tuan, Nguyen Van
Tuissi, Ausonio
Tuladhar, Rabin
Tulliani, Jean-Marc
Turner, I.G.
Turner, Chris
Ubertini, Filippo
Uddin, Ashraf
Uddin, Zakir
Ueda, Tadaharu
Uhlmann, Eckart
Ulbricht, Andreas
Umegaki, Tetsuo
Undisz, Andreas
Unosson, Erik
Upadhyay, Piyush
Ushak, Svetlana
Vaidya, Rahul
Vairo, Giuseppe
Val, Dimitri
Valberg, Henry
Valbusa, Ugo
Vallés, Cristina
Vallianatos, Filippos
Van Den Heede, P.
Vandenbussche, F.
Varin, Robert
Varlamov, Sergey
Vasdravellis, George
Vasilopoulou, Maria
Vavassori, Paolo
Vázquez, J.
Vedarajan, Raman
Velasco Ortega, E.
Venanzi, Ilaria
Venugopal, Jayarama R.
Verma, Madan
Viana da Fonseca, A.
Viegas, Diana Catarino
Vilé, Gianvito
Vindigni, Floriana
Visintin, Phillip
Vitry, Veronique
Vodenitcharova, Tania
Vogel, Florian
Vogt, Jochen
Von der Mark, Klaus
de Vos, Paul
Van Der Vurst, Farid
Wach, Radosław A.
Waclawik, Eric R.
Wadajkar, Aniket S.
Wade, Scott
Wagner, Daniel
Wahl, Siegfried
Wakabayashi, Noriyuki
Walsh, William R.
Walsh, F.C.
Wang, Yapei
Wang, Feng
Wang, Xizu
Wang, Xuefeng
Wang, Shanfeng
Wang, Jinqi
Wang, Wei
Wang, Chong-Min
Wang, Jian
Wang, Tzu-Wei
Wang, Y.
Wang, Y.H.
Wang, Jianyun
Wang, Fuke
Wang, Guofeng
Wang, Chen-Hao
Wang, Meng-Jiy
Wang, Hongfeng
Wang, Shanyong
Wang, Yuan
Wang, Xiaoming
Ward, L.P.
Ward, Liam
Wardeh, George
Wark, Michael
Wäsche, Rolf
Watauchi, Satoshi
Wauters, J.
Wei, Mei
Wei, Gang
Weil, Edward D.
Weiler, Mathias
Weimer, Jeffrey
Weinläder, Helmut
Weinstein, Dana
Weise, Jörg
Weiss, Stephan
Welker, Mark E.
Wells, Dominic
Wendel, Hans Peter
Wenner, Sigurd
West, Caroline
Westermann, Ida
White, Mary Anne
White, Shane N.
White, Joshua A.
Wiegand, Annette
Wildemann, Britt
Wilén, Carl-Eric
Wilkinson, Angus
Willander, Magnus
Wille, Kay
Williams, Dustin L.
Winterhalter, Mathias
Wiskott, H.W. Anselm
Wittmann, Folker
Wittmar, Alexandra
Wollschläger, J.
Wong, Eugene
Wong, Kenneth K.Y.
Wong, Rebecca
Worni, Andreas
Wright, Catherine
Wu, Dongjiang
Wu, Chia-Wen
Wu, G.M.
Wu, C.H.
Wu, Hsuan-Chung
Wu, Xijia
Wu, H.S.
Wu, Shin-Tson
Wu, Qinglin
Wu, Weite
Wujcik, Evan
Xiao, Boqi
Xie, Rong-Jun
Xioa, Bo
Xiong, Haifeng
Xiong, Chi
Xu, Xiaoming
Xu, Jingsan
Xu, Guo Qin
Xu, Zhijun
Xu, Haixuan
Yagoubi, J.E.
Yahiaoui, R.
Yamal-Turbay, E.
Yamamoto, Atsushi
Yamazaki, Tomohiko
Yan, Yigang
Yan, Bangbo
Yan, Liang
Yan, Haixue
Yan, Libo
Yanase, K.
Yang, Te-Hsin
Yang, Yang
Yang, Bao
Yang, Jun
Yang, Jen-Chang
Yang, Tsong-Jen
Yang, Xiaodong
Yang, Fan
Yang, Hoichang
Yang, Jinkyu
Yannopoulos, S.N.
Yao, Jianjun
Ye, Haitao
Ye, Delai
Ye, Jun
Yeh, An-Chou
Yeh, Jui-Ming
Yen, Fu-Su
Yeon, Jung Heum
Yi, Xuemei
Yildirim-Ayan, Eda
Ying, Zhining
Yoon, Ung Chan
Yoon, Seog-Young
Yoshida, Jun
Yoshida, Sanichiro
Yoshihara, Hiroshi
Yoshii, Toshitaka
Yoshinari, Masao
Yoshitake, Isamu
Yoshizawa, Satoko
You, Wei
Youn, Duck Hyun
Yousif, Belal
Yperman, Jan
Yu, Xiong
Yu, Chang-Feng
Yu, Kyeong Min
Yu, Jian
Yu, Zhengshan
Yu, Q.L.
Yu, Qingsong
Yu, Dong
Yu, Guihua
Yu, Fisher (Hua)
Yue, Zhongren
Yui, Nobuhiko
Yun, Tae
Yun, Ji Sun
Zaghi, Arash E.
Zakutayev, Andriy
Zalnezhad, Erfan
Zangle, Thomas
Zappalorto, Michele
Zaraska, Leszek
Zarbin, Marco A.
Zarnani, Pouyan
Zarogoulidis, Paul
Zehetbauer, Michael
Zerbetto, Francesco
Zeugolis, Dimitrios
Zevenhoven, Maria
Zhang, Xinli
Zhang, Chi
Zhang, Liangchi
Zhang, Qianbing
Zhang, Mingzhong
Zhang, Chengfei
Zhang, Boyu
Zhang, Yiying
Zhang, Zuhua
Zhang, Yang
Zhang, Jing
Zhao, Q.
Zhao, Jing
Zhao, Tong
Zhao, Qian
Zheng, Guang Ping
Li, Zhenhai
Zhou, Bin
Zhou, Zhengping
Zhou, Xiangming
Zhu, Shengyu
Zhu, Huayang
Zhu, Caigang
Zhu, Jiahua
Zhu, Yinghuai
Zhuiykov, Serge
Zhuo, Chuanwei
Zi, Yunlong
Ziebarth, Noël M.
Zietz, Carmen
Zijlstra, Peter
Zille, Andrea
Zinelis, Spiros
Zitoune, Redouane
Zona, Alessandro
Zucchi, Fabrizio

